# Simplified Synthesis of Isotopically Labeled 5,5-Dimethyl-pyrroline *N*-Oxide

**DOI:** 10.3390/molecules16108428

**Published:** 2011-10-10

**Authors:** Fabian Leinisch, JinJie Jiang, Leesa J. Deterding, Ronald P. Mason

**Affiliations:** 1 Laboratory of Toxicology and Pharmacology, National Institute of Environmental Health Sciences, National Institutes of Health, 111 TW Alexander Drive, Research Triangle Park, NC 27709, USA; 2 Laboratory of Structural Biology, National Institute of Environmental Health Sciences, National Institutes of Health, 111 TW Alexander Drive, Research Triangle Park, NC 27709, USA

**Keywords:** spin trap, isotopic labeling, DMPO

## Abstract

5,5-Dimethylpyrroline *N*-oxide (^15^N) and 5,5-di(trideuteromethyl)pyrroline *N*-oxide were synthesized from the respective isotopically labeled 2-nitropropane analogs obtained from the reaction of sodium nitrate with 2-halopropanes. This facile, straightforward process allows synthesizing isotopically labeled DMPO analogs in a 4-step reaction without special equipment.

## 1. Introduction

Nitrone spin traps such as the most commonly used 5,5-dimethylpyrroline *N*-oxide (DMPO) are important reagents for the detection of free radicals by means of ESR spin trapping [1]. For certain mass spectrometry experiments or to investigate the fidelity of spin trapping, it is helpful to use isotopically labeled spin traps.

The di(trideuteromethyl) analog of DMPO with its mass shift of +6 compared to unlabeled DMPO can be used for dual spin-trapping mass spectrometry experiments. Here, spin traps labeled with stable isotopes (^2^H, ^15^N or ^13^C) are utilized to simplify the interpretation of mass spectrometry experiments [2,3]. With equal amounts of the labeled and unlabeled spin trap present, adducts of the trapped radicals will appear as ion pairs in the mass spectrum (with the mass differences depending on the isotopes incorporated in the spin trap and on the charge state of the ion). This facilitates and clarifies the identification of radical-derived analytes.

The ^15^N analog can be used to unambiguously determine the susceptibility of a particular spin-trapping experiment to the Forrester-Hepburn artifact in an ESR experiment [4]. A Forrester-Hepburn artifact is the result of nucleophilic attack of the radical precursor on the spin trap with subsequent oxidation to the identical nitroxide radical as derived from genuine spin trapping. It is difficult to distinguish between nucleophilic attack and free-radical trapping, with normal chemical and biological control experiments being of no use. Timmins *et al.* reported a method based on spin traps with different isotopes at the α- or β-positions to the nitrogen of the spin trap [4]. The substrate is preincubated with a spin trap (first isotope), and then the spin trap labeled with the second isotope is added simultaneously with the initiation of free radical formation. Because the ESR signals are different, the origin of the signal can be determined as artifact or genuine signal. Employing that technique, we were able to identify the DMPO/^•^CN radical, supposedly generated by horseradish peroxidase and hydrogen peroxide, as an artifact (unpublished data).

**Scheme 1:**
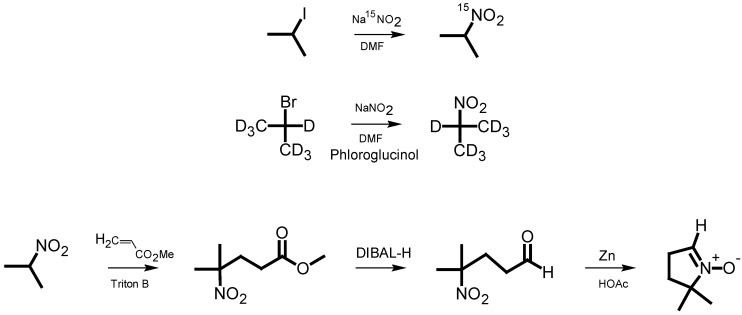
Pathway of DMPO analog synthesis.

The classical synthesis of DMPO, as reported by Janzen *et al.* [5], is based on the synthesis of pyrrolines [6]. Later, Le *et al. *[7] published a synthesis of 2-^14^C-DMPO that avoided the direct Michael reaction of nitropropane with methyl acrylate, thereby improving the low yield and eliminating the difficult purification of this step. For the synthesis of isotopically labeled DMPO, Pou *et al.* [8] published an effective method starting from ^15^N-hydroxylamine, which involved the use of hydrogen gas in an autoclave as well as a reaction with ozone derived from an ozone generator. We have developed a more facile synthetic pathway for the synthesis of DMPO based on ^15^N-sodium nitrite or 2-bromopropane (D7) as the isotopically labeled starting material. The intermediate 2-nitropropane was prepared in a one-step reaction according to the reaction principle described by Kornblum *et al.* [9]. This principle has been used for nitrone spin trap synthesis [10]. Nitropropane was then used for a DMPO synthesis similar to that of Le *et al.* [7] (Scheme 1).

## 2. Results and Discussion

### 2.1. 2-Nitropropane

The synthesis of 2-nitropropane can be accomplished directly by nitration of 2-halopropanes with sodium nitrite [9]. With 2-iodopropane, the reaction is carried out in dry DMF in the presence of urea. For the slower reacting 2-bromopropane, a longer reaction time and the presence of phloroglucinol as a nitrite ester scavenger is required.

To synthesize ^15^N-DMPO, 2-iodopropane was used in slight excess in the absence of phloroglucinol because phloroglucinol did not increase the yields with respect to the ^15^N-nitrite. The nitrite forms the nitro compound and, as a byproduct in a slow process, the nitrite ester. In an undesired reaction, the latter can react with already formed nitropropane, but the nitrite ester can be removed with phloroglucinol [9]. However, under the chosen conditions, the formation of the nitrite ester is not the limiting factor for the yield. After purification by vacuum distillation, the yield of ^15^N-2-nitropropane was 35%. Perdeuterated 2-nitropropane (D7) was synthesized from 2-bromopropane (D7) in the presence of phloroglucinol since this was more economical than employing the corresponding deuterated iodo-compound. The reaction gave perdeuteronitropropane, formed with 27% yield after purification. The yields are not very high for one-step reactions, but still result in a higher overall yield of 2-nitropropane than a multistep process (e.g., Gabriel-synthesis of the corresponding phthalate, cleavage of the 2-propylamine [11] and subsequent oxidation by ozone, which can induce isomerization).

### 2.2. Methylnitrovaleric Acid Methyl Ester

In order to avoid the reported problems of the direct aldehyde synthesis via the Michael reaction [5], we chose, instead, the conditions described by Moffett [12] to optimize the yield with respect to the nitropropane. The yield of ^15^N-nitropropane was 51% and the yield of the 1,1,1,3,3,3-hexadeutero-2-nitropropane was 80%, which was comparable to that reported in the literature [12].

### 2.3. Methylnitropentanal

The corresponding pentanal was formed by reduction with diisobutylaluminum hydride, as described by Le *et al.* [7]. Some experimental details of the protocol were modified according to [13]. In order to achieve a selective reduction to the aldehyde with minimal further reduction to the alcohol, which is difficult to remove once formed, the bath temperature should be maintained at −90 °C during the reaction. The use of wet silica gel allows the quenching procedure to be carried out at the reaction temperature [13]. The reaction gave yields of 58% for the hexadeutero- and 74% for the ^15^N-compound, which is in the expected range for diisobutylaluminum hydride reductions [13].

### 2.4. DMPO

The final step was carried out as described in the literature [7]. The yield of DMPO was 15% with both analogs. In preliminary experiments, the activation of zinc or variations of the zinc equivalents did not improve the yield to 60%, as previously reported [14]. In our hands, we obtained *ca.* 15–20% (as also reported by Le *et al. *[7]). In retrospect, for future syntheses it may be beneficial to protect the aldehyde as dioxolane before the reduction step [5] because Rosen *et al.* also reported a low yield for the direct reaction [15].

The synthesized DMPO analogs and ^14^N-DMPO were analyzed using high mass resolution mass spectrometry (Rs > 10,000). The protonated molecular ion of each analog was observed and the resulting exact mass measurement was determined. The resulting M+H^+^ ions observed for each DMPO analog were as follows: DMPO (M+H)^+^ ion of *m/z* 114.0915 (theoretical M+H^+^ = 114.0919), ^15^N-DMPO (M+H)^+^ ion of *m/z* 115.0882 (theoretical M+H^+^ = 115.0889), and ^6^D-DMPO (M+H)^+^ ion of *m/z* 120.1282 (theoretical M+H^+^ = 120.1295). According to their elemental compositions, these exact mass measurements correspond to mass accuracies of 3.5 ppm, 6.1 ppm, and 10.8 ppm, respectively. In addition, similar fragmentations of each analog were observed.

**Figure 1:**
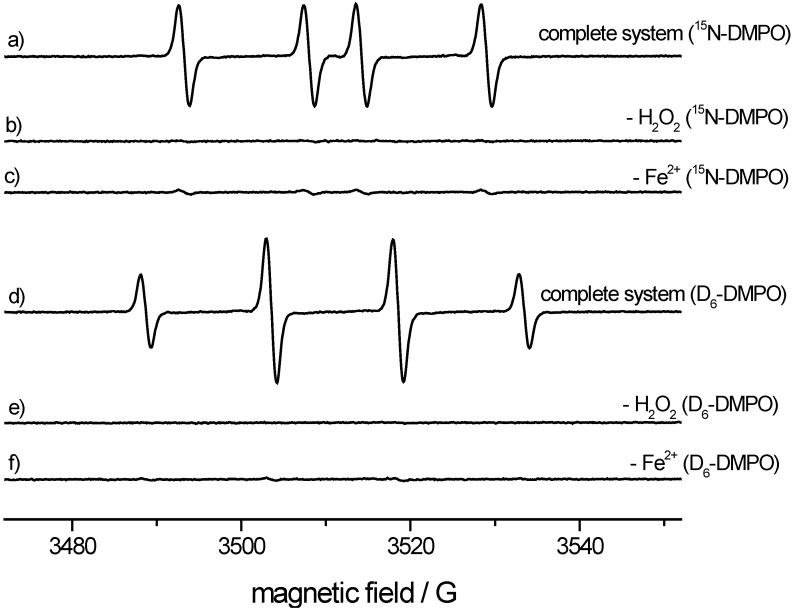
ESR spin trapping of hydroxyl radicals with 100 mM ^15^N- or di(trideuteromethyl) DMPO. Radicals were generated with a Fenton System (100 μM FeSO_4_, 200 μM H_2_O_2_ in 100 mM phosphate buffer, pH 7.4, with 200 μM DTPA). A strong signal was detected with ^15^N-DMPO (**a**) as well as with di(trideuteromethyl) DMPO (**d**). No significant signal was detected without H_2_O_2_ (**b** and **e**) or Fe^2+^ (**c** and **f**).

With a spin-trapping experiment, the coupling constants of the respective hydroxyl radical adducts were determined. A strong signal was detected with both analogs (Figure 1a,d). With the di(trideuteromethyl) analog, a signal with similar coupling constants and the same intensity as with ^14^N-DMPO emerged (D6-DMPO: a^N^ = 14.97 G and a^H^ = 14.77 G; ^14^N-DMPO: a^N^ = 14.96 G and a^H^ = 14.76 G, not shown. Literature: a^N^ = 14.8 and a^H^ = 14,8 [8]). With the ^15^N-analog, coupling constants were determined to be a^N^ = 20.95 G and a^H^ = 14.78 G. The change of the nitrogen hyperfine coupling constant is due to the gyromagnetic ratio of ^15^N:^14^N = 1.40 [16,17]. In the absence of hydrogen peroxide (b and e) or iron (c and f), no significant signal was detected with either analog. 

## 3. Experimental

^15^N-NaNO_2_ and 2-bromopropane (D7) were purchased from Cambridge Isotope Laboratories (Andover, MA, USA). Phloroglucinol was purchased from Alfa Aesar (Ward Hill, MA, USA). ^14^N-DMPO was purchased from Dojindo (Rockville, MD, USA). Other chemicals were purchased from Sigma-Aldrich (St. Louis, MO, USA). Where dry solvents were required, the glassware was oven-dried and a dry argon atmosphere was used. These solvents were purchased as ‘anhydrous’ grade and handled under air exclusion. ESR spectra were recorded with a Bruker ElexSys E-500 spectrometer with ER 4122SHQ cavity, flow injection electrospray ionization (ESI/MS) analyses were performed with a Waters Q-TOF Premier mass spectrometer. ^1^H NMR spectra were acquired at 25 °C on a Varian INOVA 600 spectrometer operating at ^1^H frequency of 599.763 MHz.

### 3.1. Synthesis of ^15^N-5,5-Dimethyl-1-pyrroline N-oxide

*^15^N-2-Nitropropane.* In a 500 mL three-neck flask equipped with a magnetic stir bar, septum, Ar supply and a gas bubbler, urea (17 g, 0.28 mol) was dissolved in dry N,N-dimethylformamide (250 mL) under an Ar atmosphere. Then, ^15^N-NaNO_2_ (10 g, 145 mmol) was added and allowed to dissolve. 2-Iodopropane (18 mL; 180 mmol) was added and the solution was stirred for 5 h at room temperature. The reaction was quenched by pouring the reaction mixture into ice water (300 mL) with diethyl ether (100 mL). The organic layer was separated and retained, and the aqueous layer was extracted four times with diethyl ether. The combined organic layers were washed twice with sodium thiosulfate solution (10 wt%, 25 mL) followed by washing with saturated sodium bicarbonate solution (2 × 100 mL). The organic layer was dried over anhydrous magnesium sulfate, filtered, and the solvent removed with a rotary evaporator to obtain the crude product. The product was purified by vacuum distillation (−80 kPa, 75 °C) to give 4.63 g (51 mmol, 35%) of ^15^N-2-nitropropane. ^1^H-NMR (CDCl_3_): 1.51 (m, 6H), 4.62 (m, 1H).

*^15^N-4-Methyl-4-nitropentanoic acid methyl ester.* To a 100 mL three-neck flask with magnetic stir bar, condenser and addition funnel, ^15^N-2-nitropropane (4.63 g, 51 mmol), 1,4-dioxane (5 mL) and an aqueous solution of benzyltrimethylammonium hydroxide (Triton B, 40%, 1 mL) were added*.* The contents were heated to 70 °C (water bath) with stirring, and methyl acrylate (4.5 mL, 52 mmol) was added dropwise over 15 min. The temperature was raised and the solution stirred at 85 °C for 3 h. After cooling to room temperature, the solution was diluted (100 mL of H_2_O) and acidified with diluted hydrochloric acid (1 M). The reaction mixture was then extracted five times with diethyl ether (100 mL each). The combined organic layers were washed with water, saturated sodium bicarbonate solution and water again (100 mL each), then dried over anhydrous NaSO_4_. The solvent was removed on a rotary evaporator to give the crude product, which was purified by column chromatography on silica gel (hexane:diethyl ether = 1:1) to give 4.62 g (26.4 mmol, 51%) of ^15^N-4-methyl-4-nitropentanoic acid methyl ester. An r_f_ of 0.33 was found. The product was stored under an argon atmosphere. ^1^H-NMR (CDCl_3_): 1.57 (s, 3H), 1.78 (s, 3H), 2.26 (m, 2H), 2.32 (m, 2H), 3.67 (s, 3H).

*^15^N-4-Nitro-4-methyl-1-pentanal.* A 500 mL three-neck flask equipped with a magnetic stir bar, septum, Ar supply and gas bubbler was filled with dry dichloromethane (125 mL) with the exclusion of air. ^15^N-4-methyl-4-nitropentanoic acid methyl ester (4.62 g; 26.4 mmol) was added, and the solution was cooled down to −95 °C with a methanol bath using dry ice initially, then liquid nitrogen for temperature adjustment. Next, 1 M diisobutylaluminum hydride solution in dichloromethane (29 mL) was added dropwise over 5 min, keeping the temperature below −90 °C. After 40 min of stirring at −95 °C, the solution was quenched by the addition of wet silica gel (20 g of silica gel well-mixed with 10 mL of H_2_O) while maintaining the reaction mixture at −95 °C. After 10 min of stirring at −95 °C, the cold bath was removed and the mixture was allowed to equilibrate to room temperature. The silica gel was separated by filtration and extracted three times with dichloromethane (100 mL each portion). The combined organic layers were washed with dilute hydrochloric acid, saturated sodium bicarbonate solution and water (100 mL each) and dried over anhydrous sodium sulfate. The removal of dichloromethane with a rotary evaporator gave the raw product, which was purified by silica gel flash chromatography (ethyl acetate:hexane = 1:5) to give 2.85 g (19.2 mmol, 74% yield) of ^15^N-4-nitro-4-methyl-1-pentanal. The r_f_ value was determined to be 0.29. The product was stored under argon at −20 °C. ^1^H-NMR (CDCl_3_): 1.58 (m, 6H), 2.22 (bt, 2H), 2.49 (t, *J *= 7.2 Hz, 2H), 9.75 (s, 1H).

*^15^N-5,5-Dimethyl-1-pyrroline N-oxide.* In a 500 mL three-neck flask with an addition funnel, ethanol (95%, 60 mL) was cooled to 2 °C. Then ^15^N-4-nitro-4-methyl-1-pentanal (2.85 g, 19.2 mmol) and zinc dust (2.68 g, 0.41 mmol, 2.1 equivalents) were added. Under brisk magnetic stirring, glacial acetic acid (4.2 mL) was added dropwise while the temperature was kept below 10 °C. After stirring 1 h, the apparatus was put in a refrigerator suitable for flammable materials. Any hydrogen gas which may have formed was allowed to dissipate. After an additional 30 h of stirring at 10 °C, the solution was filtered to remove zinc and any undissolved zinc acetate. The filtrate was extracted with cold ethanol (3 × 100 mL). From the combined solutions, the ethanol was removed with a rotational evaporator. The residue was diluted with dichloromethane (200 mL) and washed twice with saturated sodium bicarbonate solution and water (50 mL each). The organic layer was dried with magnesium sulfate, filtered and the dichloromethane removed with a rotary evaporator to give a reddish-brown crude product. The product was purified by two consecutive sublimations (0.1 torr) from room temperature to 0 °C, and 305 mg (2.7 mmol, 15%) of ^15^N-DMPO was obtained. ^1^H-NMR (CDCl_3_): 1.42 (m, 6H), 2.12 (td, 1.5 Hz, 7.6 Hz, 2H), 2.56 (m, 2H), 6.80 (t, *J *= 2.6 Hz, 1H).

### 3.2. Synthesis of 5,5-Di-(trideuteromethyl)-1-pyrroline N-oxide

*2-Nitropropane (D7).* Urea (26.5 g, 0.44 mol), and phloroglucinol (21 g, 0.17 mol) were dissolved in dry *N*,*N*-dimethylformamide (600 mL) in a 1000 mL three-neck flask equipped with a magnetic stir bar, septum, Ar supply and bubbler, under an Ar atmosphere. Then, NaNO_2_ (24.74 g, 0.36 mol) was added and dissolved. Heptadeutero-2-bromopropane (20 g, 154 mmol) was added, and the solution was stirred for 48 h at room temperature. The reaction was quenched with ice water (1000 mL) covered with diethyl ether (200 mL). The organic layer was separated and collected, and the aqueous layer was extracted four times with diethyl ether (100 mL each). The combined organic layers were washed with saturated sodium bicarbonate solution (100 mL) and water (2 × 100 mL). The organic layer was dried over anhydrous magnesium sulfate and filtered. The solvent was removed with a rotational evaporator to obtain the crude product. The product was purified by vacuum distillation (−80 kPa, 75 °C) to give 4.02 g (42 mmol, 27%) of 2-nitropropane (D7). 

*4,4,4-Trideuteromethyl-4-nitro-5,5,5-trideuteropentanoic acid methyl ester. *The ester was synthesized from 2-nitropropane (D7) (4.02 g, 42 mmol), dioxane (5 mL), Triton B (1 mL) and methyl acrylate (4.1 mL) as described earlier for ^15^N-4-methyl-4-nitropentanoic acid methyl ester. The yield was 6.11 g (33.72 mmol, 80%) after column chromatography (hexane:diethyl ether = 4:1) and the removal of the solvent. ^1^H-NMR (CDCl_3_): 2.26 (m, 2H), 2.32 (m, 2H), 3.67 (s, 3H).

*4-Nitro-4,4,4-trideuteromethyl-5,5,5-trideutero-1-pentanal.* The aldehyde was synthesized as described for ^15^N-4-nitro-4-methyl-1-pentanal with dry dichloromethane (200 mL), the deuterated nitromethylpentanoic acid methyl ester (6.11 g, 33.72 mmol) and a 1 M stock solution of diisobutylaluminum hydride (37.1 mL) using water-free chemicals under an argon atmosphere. After flash chromatography (hexane:ethyl acetate = 5:1) and removal of the solvent, 2.86 g (19.67 mmol, 58%) of the pure product was obtained. ^1^H-NMR (CDCl_3_): 2.14 (t, *J *= 7.4 Hz, 2H), 2.42 (t, 7.4 Hz, 2H), 9.67 (s, 1H).

*5,5-Di(trideuteromethyl)-1-pyrroline N-oxide.* The DMPO analog was synthesized similarly to ^15^N-5,5-dimethyl-1-pyrroline *N*-oxide, using ethanol (100 mL), the aldehyde intermediate (4.08 g; 19.67 mmol), zinc dust (3.69 g, 56.43 mmol, 2.9 eq) and acetic acid (6.75 g, 112.4 mmol). Purification gave 357 mg (2.75 mmol) of colorless DMPO crystals (14% yield). ^1^H-NMR (CDCl_3_): 2.12 (td, 1.5 Hz, 7.6 Hz, 2H), 2.56 (td, 2.5 Hz, 7.4 Hz, 2H), 6.82 (t, *J *= 2.5 Hz, 1H).

*ESR spectroscopy*. We used a microwave frequency of 9.78 GHz (X-Band), a microwave power of 20 mW and 1 × 10^4^ receiver gain, a modulation frequency of 100 kHz and modulation amplitude of 1.0 G. Time constant and conversion time were 40.96 ms. The field range was 80 G with a center field of 3512 G. For the spin-trapping experiment, 100 mM of the ^15^N- or di(trideuteromethyl)DMPO analog was mixed with 100 μM FeSO_4_, 200 μM diethylenetriaminepentaacetic acid and 200 μM hydrogen peroxide. The phosphate buffer (100 mM, pH = 7.4) was treated with Chelex-100 to reduce metal contamination. In control experiments, the iron or H_2_O_2_ was omitted for comparison. The experiment was repeated with regular ^14^N-DMPO.

*Mass Spectrometry*. Samples of the DMPO analogs were initially prepared at 100 mM in acetonitrile, diluted 1000-fold just prior to analysis with a solution of water with 0.1% formic acid and infused into the mass spectrometer at 500 nL/min using a syringe pump. The instrumental parameters for the MS analyses were as follows: capillary voltage, 3.5 kV; cone voltage, 30 V; collision energy, 4 eV and source temperature, 80 ºC. MS/MS data of the DMPO analogs were acquired using collision energies of 15–25 eV. For calibration, a solution of glu-fibrinopeptide B (500 fmol/μL) in water/acetonitrile 80:20 (v/v) with 0.1% formic acid and a mass of 785.8496 (2+) was used. Data analysis was accomplished using MassLynx software supplied by the manufacturer.

## 4. Conclusions

Here we describe a synthesis pathway for the isotopically labeled spin traps ^15^N-5,5-dimethyl-1-pyrroline *N*-oxide and 5,5-di(trideuteromethyl)-1-pyrroline *N*-oxide. Though syntheses for isotopically labeled DMPO have already been reported [8], the elimination of hydrogen gas and ozone as reactants and the respective special equipment facilitates the synthesis and gives reasonable yields. As reported previously [7], Michael reaction to the methyl ester and subsequent reduction to the aldehyde allows for better yields and easier purification compared to direct aldehyde formation. For reduction to DMPO, it may be beneficial to protect the aldehyde as the corresponding dioxolane first since the yield of the direct reaction was below the yield reported in the original paper. This issue has also been reported elsewhere [15]. Our approach is well suited for the synthesis of isotopically labeled DMPO analogs.
